# Supplementary material for: The NLRP6 inflammasome is activated by sterile or pathogen-induced endolysosomal damage
Source: EMBO J. 2025 Nov 20;45(1):30–63. doi: 10.1038/s44318-025-00637-4 (PMC12759077; doi:10.1038/s44318-025-00637-4)
Supplement: Supplementary file 1 — Appendix [file 44318_2025_637_MOESM1_ESM.pdf]

Appendix for:

The NLRP6 inflammasome is activated by sterile or pathogen-induced endolysosomal damage

Appendix

Table of contents:

Appendix Figure S1	page 2
Appendix Figure S2	page 4
Appendix Figure S3	page 5
Appendix Table S1	page 6
Appendix Table S2	page 7
Appendix Table S3	page 7

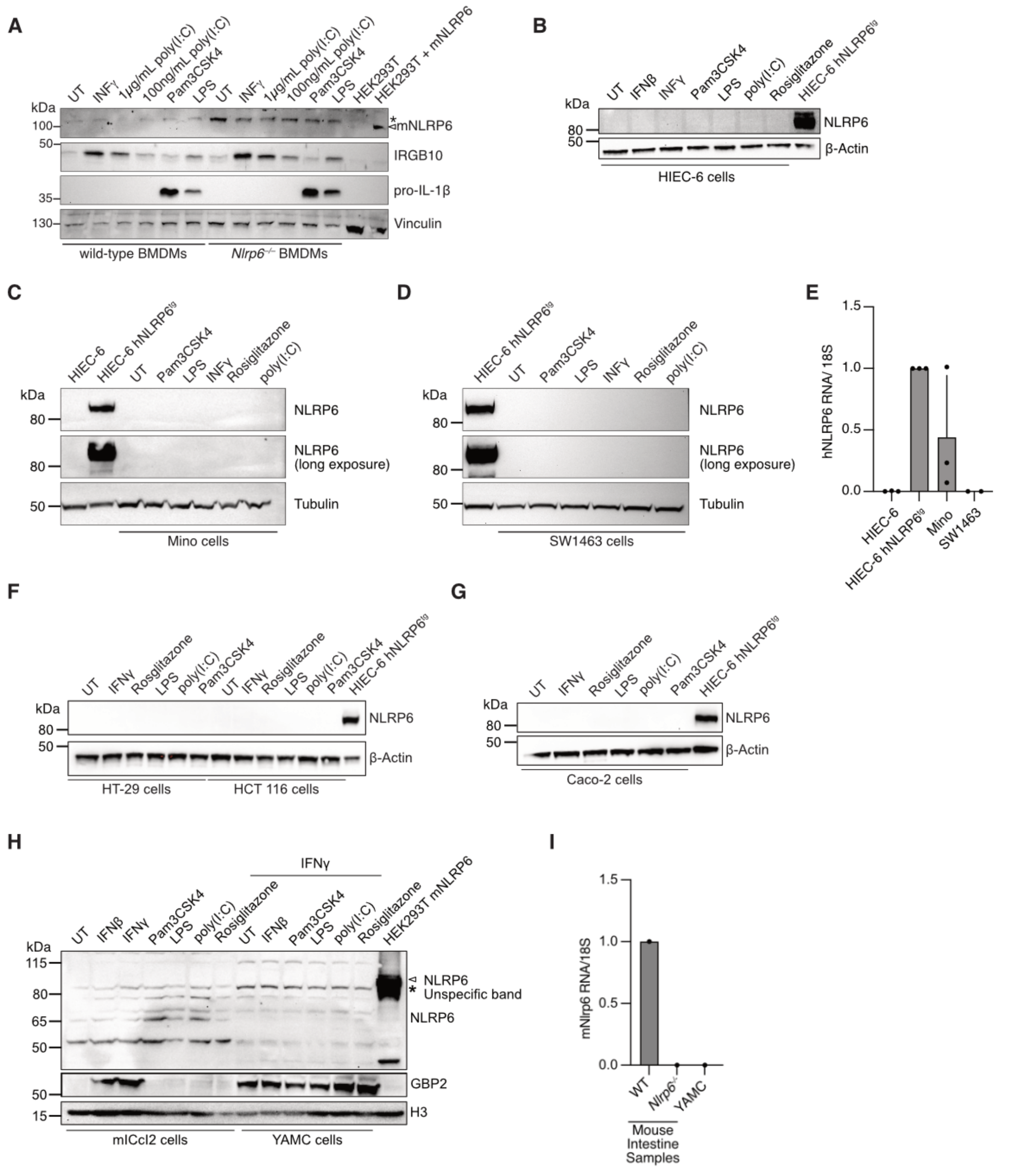

Appendix Figure S1. NLRP6 is not expressed in primary mouse macrophages or a panel of human or murine intestinal epithelial cell lines

A. Immunoblotting for NLRP6 expression in murine wild-type or *Nlrp6*−/− BMDMs primed overnight as indicated. mNLRP6-FLAG expressing HEK293T cells are used as a positive control. To check successful priming, IRGB10 and pro-IL-1β expression was used. Arrowhead indicates specific mNLRP6 band, asterisk indicates non-specific band. Vinculin is used as a loading control. **B-D.** Immunoblotting for NLRP6 expression in HIEC-6, Mino and SW1463 cell lines primed overnight as indicated. HIEC-6 transgenically expressing NLRP6 (HIEC-6 hNLRP6tg) are used as a positive control and β–actin or Tubulin are used as a loading control. **E.** qPCR analysis of hNLRP6 mRNA in HIEC-6, Mino and SW1463 cell lines. RNA isolated from HIEC-6 hNLRP6tg is used as a positive control. **F-G.** Immunoblotting for NLRP6 expression in HT-29, HCT 116 and Caco-2 cell lines primed overnight as

indicated. HIEC-6 hNLRP6^{tg} are used as a positive control. β-Actin is used as a loading control. **H.** Immunoblotting for NLRP6 expression in mICcl2 and YAMC murine intestinal epithelial cell lines primed overnight as indicated. GBP2 expression shows the successful priming by IFNγ and IFNβ. H3 is used as a loading control. Arrowhead indicates specific mNLRP6 band, asterisk indicates non-specific band. mNLRP6-FLAG expressing HEK293T cells are used as positive control. **I.** Preliminary data of qPCR analysis of mNlrp6 mRNA in YAMC mouse intestinal epithelial cells. RNA isolated from primary intestinal samples from WT and *Nlrp6*$^{-/-}$ mice are used as a positive and negative control respectively. The following concentrations were used for priming: 100ng/ml IFNβ, 10ng/ml IFNγ, 300ng/mL Pam3CSK4, 100ng/mL LPS, 100ng/mL poly(I:C), 100μM rosiglitazone. All immunoblots are representative of 2-3 independent experiments. qPCR data is from 1-3 independent experiments.

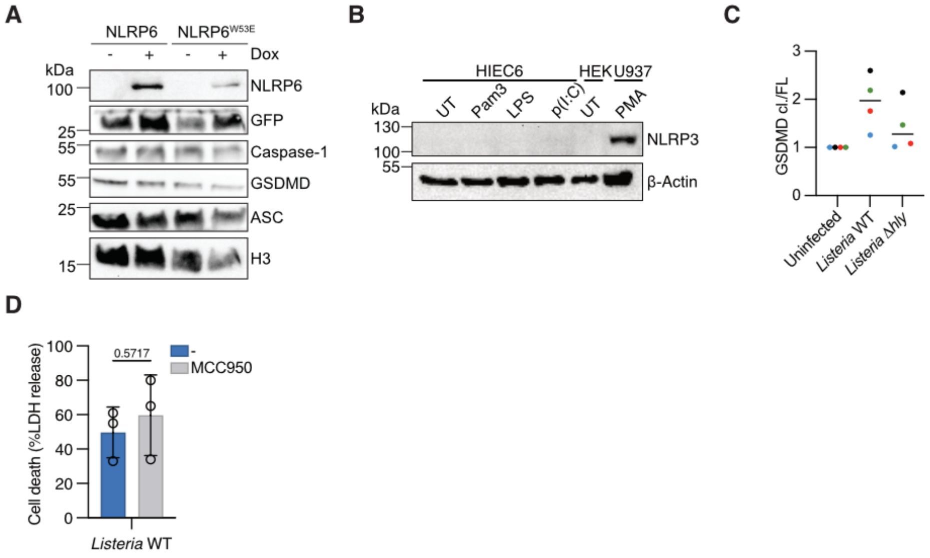

Appendix Figure S2. Expression of NLRP6 and other inflammasome components in HIEC-6 IECs. **A.** Immunoblots showing the expression of NLRP6, GFP, Caspase-1, GSDMD and ASC in NLRP6-WT^{tg} or NLRP6-W53E^{tg} HIEC-6 left untreated or treated with 1μg/mL doxycycline overnight. Expression of GFP is shown as IRES-GFP was used as a marker to select the cells by flow cytometry. H3 serves as loading control. **B.** Immunoblots showing expression of NLRP3 in HIEC-6 primed with 300ng/mL Pam3CSK4 (Pam3), 100ng/mL LPS or poly(I:C), HEK293T cells and U937 macrophages differentiated with 100ng/ml PMA. Actin serves as loading control, UT: untreated. **C.** Quantification of GSDMD cleavage from Western blots of NLRP6-WT^{tg} HIEC-6 induced 1μg/mL doxycycline overnight and then infected, or not, with *Listeria* for 8h. Each color represents one biological replicate. Line represents the median. **D.** LDH release from NLRP6-WT^{tg} HIEC-6 cells induced or not with 1μg/ml doxycycline overnight, treated or not with 10μM MCC950 30min before and during infection with WT *L. monocytogenes* EGD for 8h. Data are presented as mean ± SD of 3 independent experiments, each with three technical replicates, each data point represents the mean of one experiment. Data were analyzed by Welch's t test. Immunoblots are representative of three independent experiments.

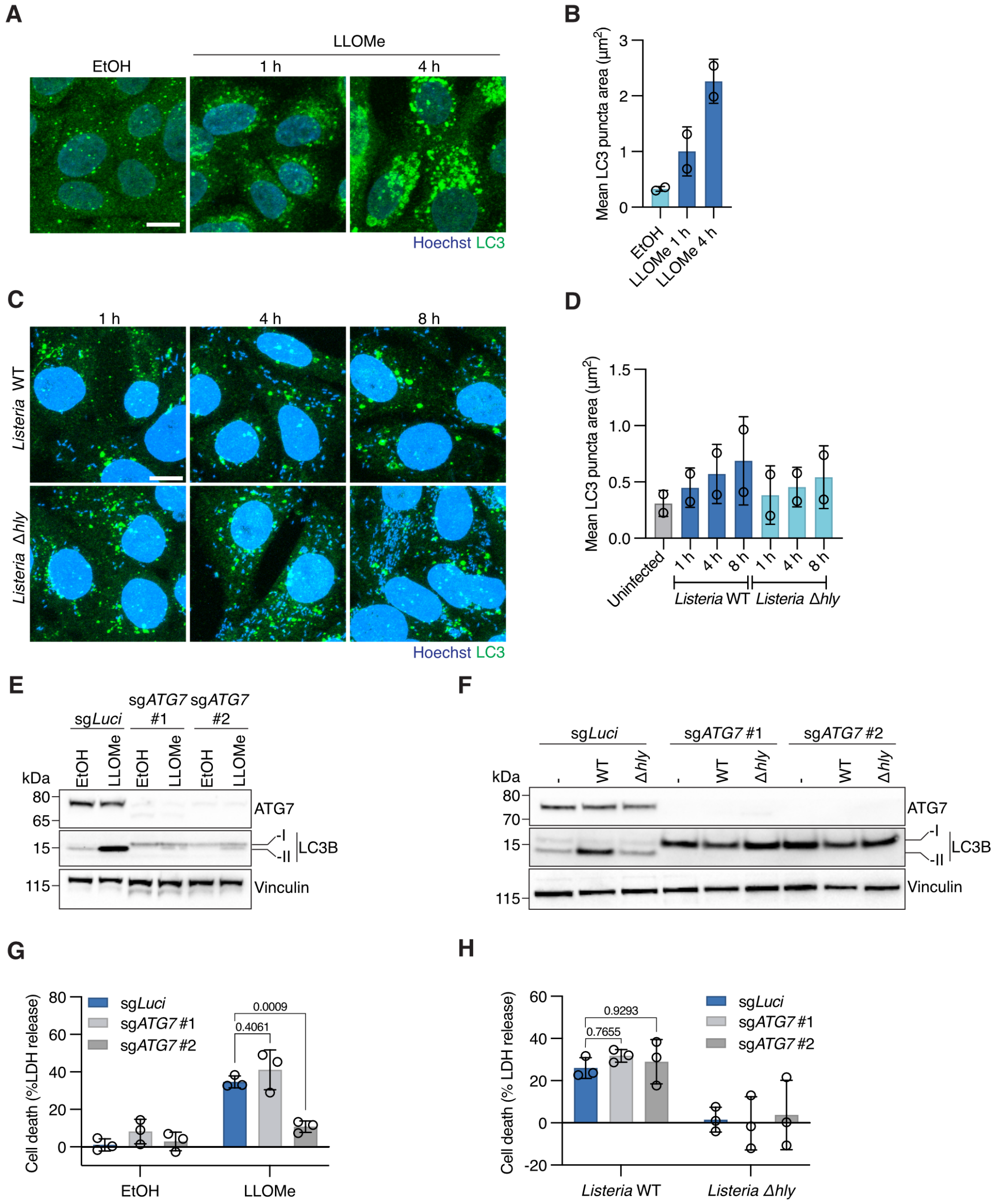

Appendix Figure S3. NLRP6 activation is independent of autophagy.

A-B. Representative maximum projection confocal micrographs and quantification of LC3 puncta area of HIEC-6 cells treated with vehicle control (ethanol, EtOH) for 4h or 0.5mM LLOMe for 1h or 4h and stained for LC3 by immunofluorescence. **C-D.** Representative maximum projection confocal micrographs and quantification of LC3 puncta area of NLRP6^{tg} HIEC-6 cells induced with 1µg/ml doxycycline overnight and infected with *L. monocytogenes* EGD WT or Δ*hly* mutant for 1, 4 or 8h and stained for LC3 by immunofluorescence. **E-F.** ATG7 expression and LC3 lipidation of NLRP6-WT^{tg} HIEC-6 controls cells (sg*Luci*) or polyclonal populations lacking ATG7 (sg*ATG7* #1 and #2) induced with 1µg/ml doxycycline overnight, either treated with 0.5mM LLOMe or an equivalent volume of ethanol (EtOH) for 4h or infected with *L. monocytogenes* EGD WT or Δ*hly* mutant for 8h. Vinculin was used as a loading control. **G-H.** LDH release from NLRP6-WT^{tg} HIEC-6 controls cells (sg*Luci*) or polyclonal populations lacking ATG7 (sg*ATG7* #1 and #2) induced with 1µg/ml doxycycline overnight and treated with 0.5mM LLOMe or equivalent volume ethanol (EtOH) for 4h or infected with *L. monocytogenes* EGD WT or Δ*hly* mutant for 8h. Graphs B and D show mean ± SD from two pooled experiments with 6 fields of view and at least 200 cells analyzed per condition and experiment. Each data point represents the mean of one experiment. Graph G and H show mean ± SD of two or three independent experiments with three technical replicates each respectively. Each data point represents the mean of one

experiment. Images and blots are representative of at least two independent experiments. Scale bars represent 5μm. Statistics used: two-way ANOVA with Tukey's multiple comparisons.

**Plasmid**	**Primers**
pANB011 mCherry-ASC	GGCTAGCGCTACCGGTgccaccatggtgagcaagggc<br>CCTCCACCcttgtacagctcgtccatgcc<br>gtacaagGGTGGAGGCGGTAGCG<br>AGTCCGGACTTGTACATTAGCTCCGCTCCAGGTCC
hNLRP6^{W53E}	CATCCCGGAGGGGCGGCTGGAGCGCGC<br>CGCCCCTCCGGGATGCTGCGTCCGTCC
hNLRP6$^{WA/WB}$	ATGGCCGCGGCGATGCCCGCCGGGCCCTG<br>CATCGCCGCGGCCATGGCGGCCAAAAAGATCCTGTAC<br>GGCCGGCGCGGCCGCGCTGCCGGCGCTGGGGGGC<br>GCGGCCGCGCCGGCCAGGATGAAGAGCAGCCGC
hNLRP6$^{\Delta FISNA(108-129)}$	TCGGGCTCCACGCTCGGGTGAAGGAGAG<br>GAGCGTGGAGCCCGAGCCGCTGCAG
hNLRP6 $^{\Delta FISNA(108-1193)}$	TCGGGCTCCGGCCGCTGACCGTGGTG<br>GCGGCCGGAGCCCGAGCCGCTGCAG
hNLRP6$^{FISNA(4A)}$	CGCGGCGGCGTACGCGGAGCACGTGCTGCAGCTG<br>GCGTACGCCGCCGCGTACTCGGACACGGAGAGCAG
hNLRP6 pINDUCER21	cctccgcggccccgaactagATGGACCAGCCAGAGGC<br>ggacgtcgtatgggtattcgTCAGAAGGTCGAGATGAGTTCCTTGG
hNLRP6^{W53E} pINDUCER21	cctccgcggccccgaactagATGGACCAGCCAGAGGC<br>ggacgtcgtatgggtattcgTCAGAAGGTCGAGATGAGTTCCTTGG
pANB018 hNLRP6-mCherry	CGCTAGCGCTACCGGTATGGACCAGCCAGAGGCC<br>CCTGATCCTGAGCCTGATCCGAAGGTCGagatgag<br>AGGCTCAGGATCAGGCTCTatggtgagcaagggc<br>GAGGTCGAGAATTCGAATTActtgtacagctcgtccatgc
pANB045 hNLRP3-mCherry	CGCTAGCGCTACCGGTatggcaagcacccgc<br>CCTGATCCccaagaaggctcaaagacgacg<br>ttcttggGGATCAGGCTCAGGATCAGGC<br>GAGGTCGAGAATTCGAATTActtgtacagctcgtccatgc
pLL01 NLRP6$^{PYD(NLRP3)}$	TAGCGCTACCGGTatgaagatggcaagcaccc<br>CCGAGCCGccacttcggctcatctcttttgc<br>gaagtggCGGCTCGGGCTCG<br>CCTGATCCTGAGCCTGATCCGAAGGTCGAGATGAG<br>AGGCTCAGGATCAGGCTCTatggtgagcaagggc<br>GAGGTCGAGAATTCGAATTActtgtacagctcgtccatgc
pLL02 NLRP6$^{PYD-linker(NLRP3)}$	TAGCGCTACCGGTatgaagatggcaagcaccc<br>TTCTTCTTgtaatctttcttcattttacaaatagagattctcgaaaggtactcc<br>agattacAAGAAGAAGTACCGGGAGCAC<br>CCTGATCCTGAGCCTGATCCGAAGGTCGAGATGAG<br>AGGCTCAGGATCAGGCTCTatggtgagcaagggc<br>GAGGTCGAGAATTCGAATTActtgtacagctcgtccatgc
pLL03 NLRP6$^{PYD-linker-FISNA(NLRP3)}$	TAGCGCTACCGGTatgaagatggcaagcaccc<br>ACGGTCAGcacaggctcagaatgctcatca<br>gcctgtgCTGACCGTGGTGCTGC<br>CCTGATCCTGAGCCTGATCCGAAGGTCGAGATGAG<br>AGGCTCAGGATCAGGCTCTatggtgagcaagggc<br>GAGGTCGAGAATTCGAATTActtgtacagctcgtccatgc
pLL16 NLRP3$^{NACHT(NLRP6)}$	TAGCGCTACCGGTatgaagatggcaagcaccc<br>ACCACGGTgtgcacaggctc<br>tgtgcacACCGTGGTGCTGCAGG<br>acccgatgGCAGCACCTCACGCA<br>GTGCTGCcatcgggtggagtcactgt<br>CCTGATCCccaagaaggctcaaagacgacg<br>AGGCTCAGGATCAGGCTCTatggtgagcaagggc

	GAGGTCGAGAATTCGAATTActtgtacagctcgtccatgc
pLL17 NLRP3$^{LRR(NLRP6)}$	TAGCGCTACCGGTatgaagatggcaagcaccc<br>CCAGCAGGacagttctcaatgcaaaaggaagaaacc<br>gaactgtCCTGCTGGACAGGCACT<br>CCTGATCCTGAGCCTGATCCGAAGGTCGAGATGAG<br>AGGCTCAGGATCAGGCTCTatggtgagcaagggc<br>GAGGTCGAGAATTCGAATTActtgtacagctcgtccatgc

Appendix Table S1. Primers used in this study to clone indicated plasmids.

Target	Sequence	Exon targeted
CASP1	GGAGCTGAGGTTGACATCAC	5
CASP4 #1	AGGGATTCCAACACCTTAAG	2
CASP4 #2	CAGTTCCGCAGATTCCCTCC	5
ATG7 #1	AAAGCAACAACATACCACAC	14
ATG7 #2	CTTGAAAGACTCGAGTGTGT	6
GSDMD	GAGGTTGACACACTTATAACG	3

Appendix Table S2. Single guide RNAs used in this study to create population wide knock-out using CRISPR-Cas9.

Target	Sequence
hNLRP6 fw	TGGCTGTTCTGAGCTACTGC
hNLRP6 rv	GTGCCTTGAGAACTGCTGC
mNlrp6 fw	CTGAGACTGGTGAGCTGTGG
mNlrp6 rv	TGGTGCTTTGAGAGCCCTTC
18S fw	GTAACCGTTGAACCCCATT
18S rv	CCATCCAATCGGTAGTAGCG

Appendix Table S3. qPCR primers used in this study to probe for gene expression
