# Supplementary figures and images for: The NLRP6 inflammasome is activated by sterile or pathogen-induced endolysosomal damage
Source: EMBO J. 2025 Nov 20;45(1):30–63. doi: 10.1038/s44318-025-00637-4 (PMC12759077; doi:10.1038/s44318-025-00637-4)

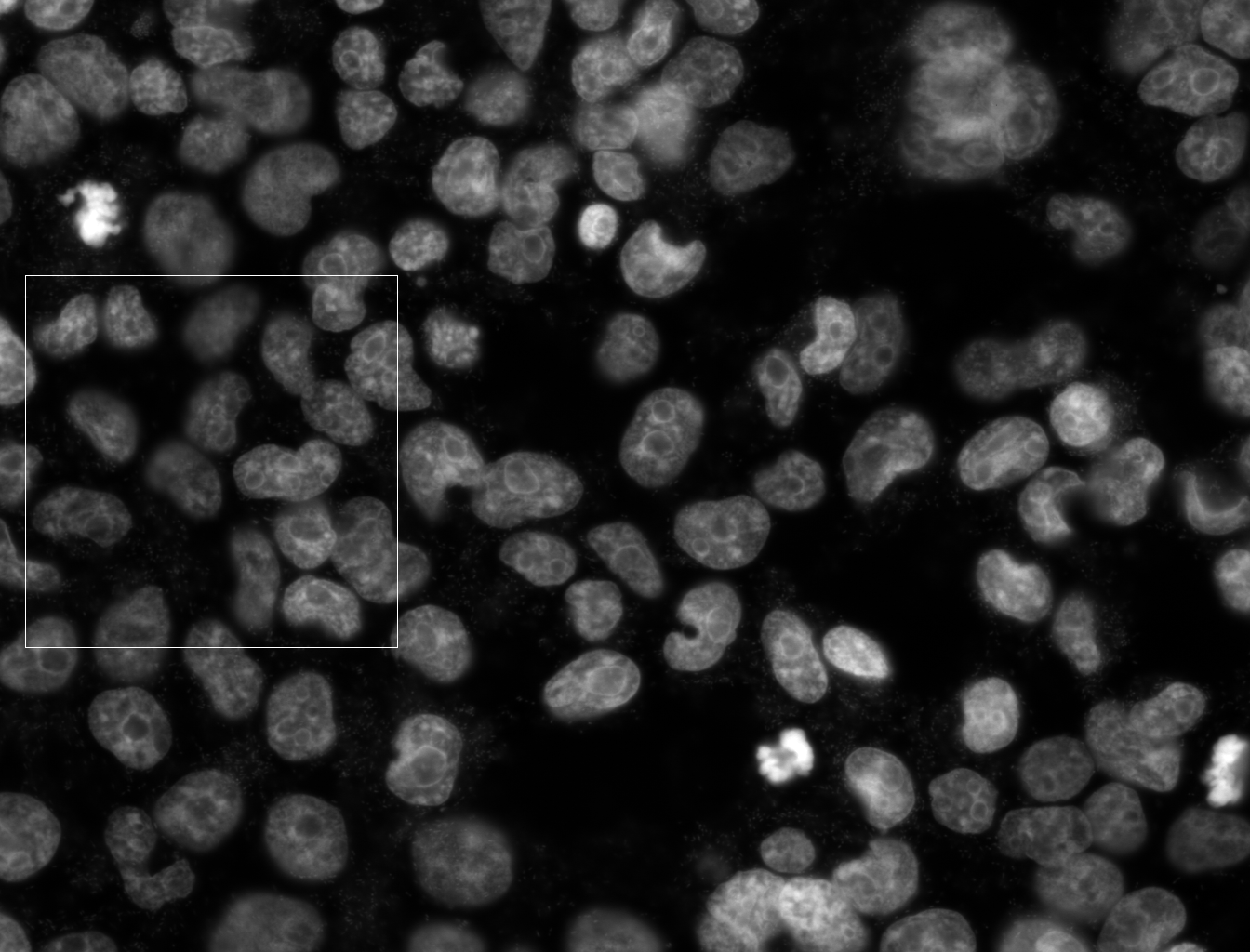

Supplement: Supplementary file 3 — Source data Fig. 1 [file 44318_2025_637_MOESM3_ESM.zip › Figure 1/1A/Listeria WT.tif]

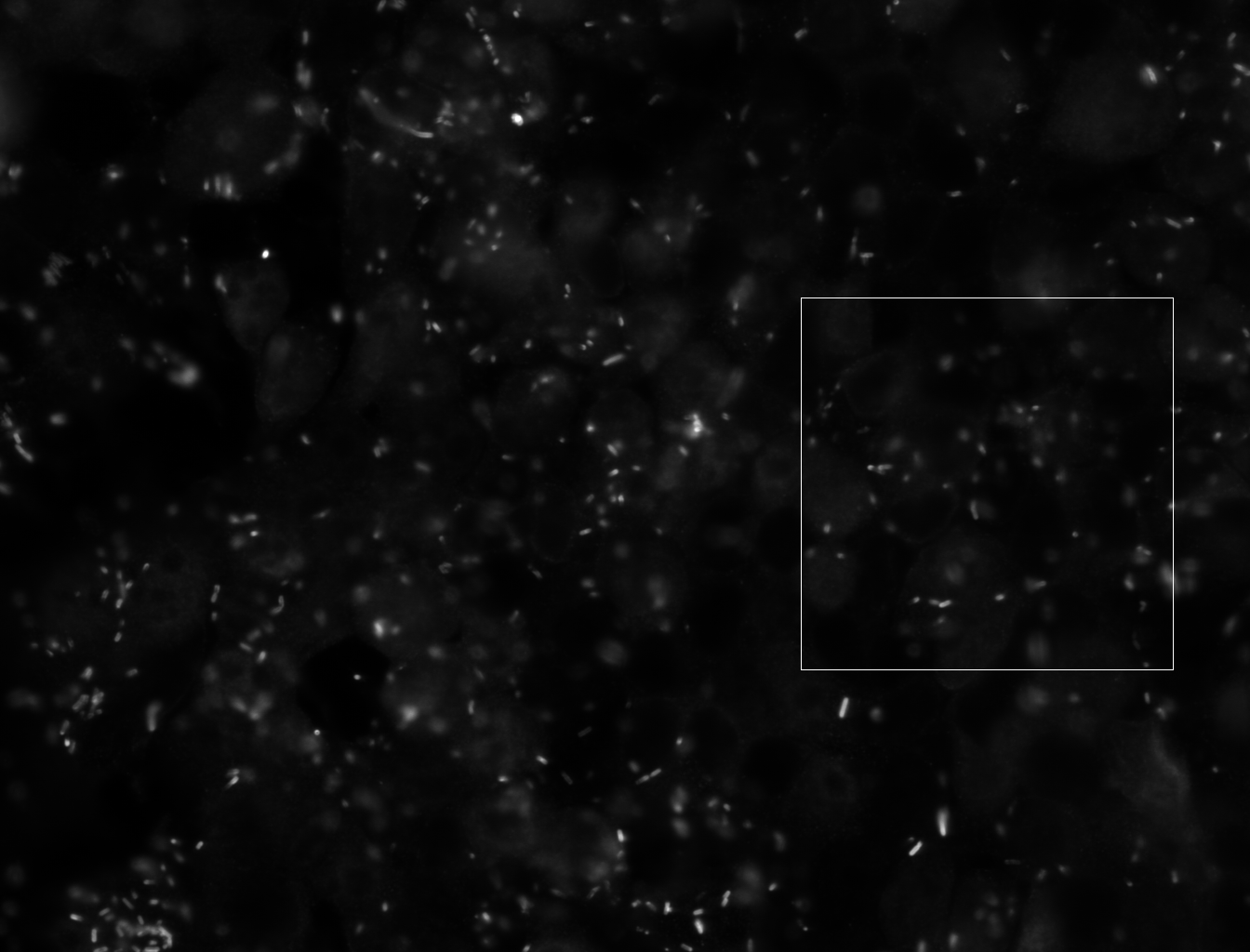

Supplement: Supplementary file 3 — Source data Fig. 1 [file 44318_2025_637_MOESM3_ESM.zip › Figure 1/1A/Listeria hly.tif]

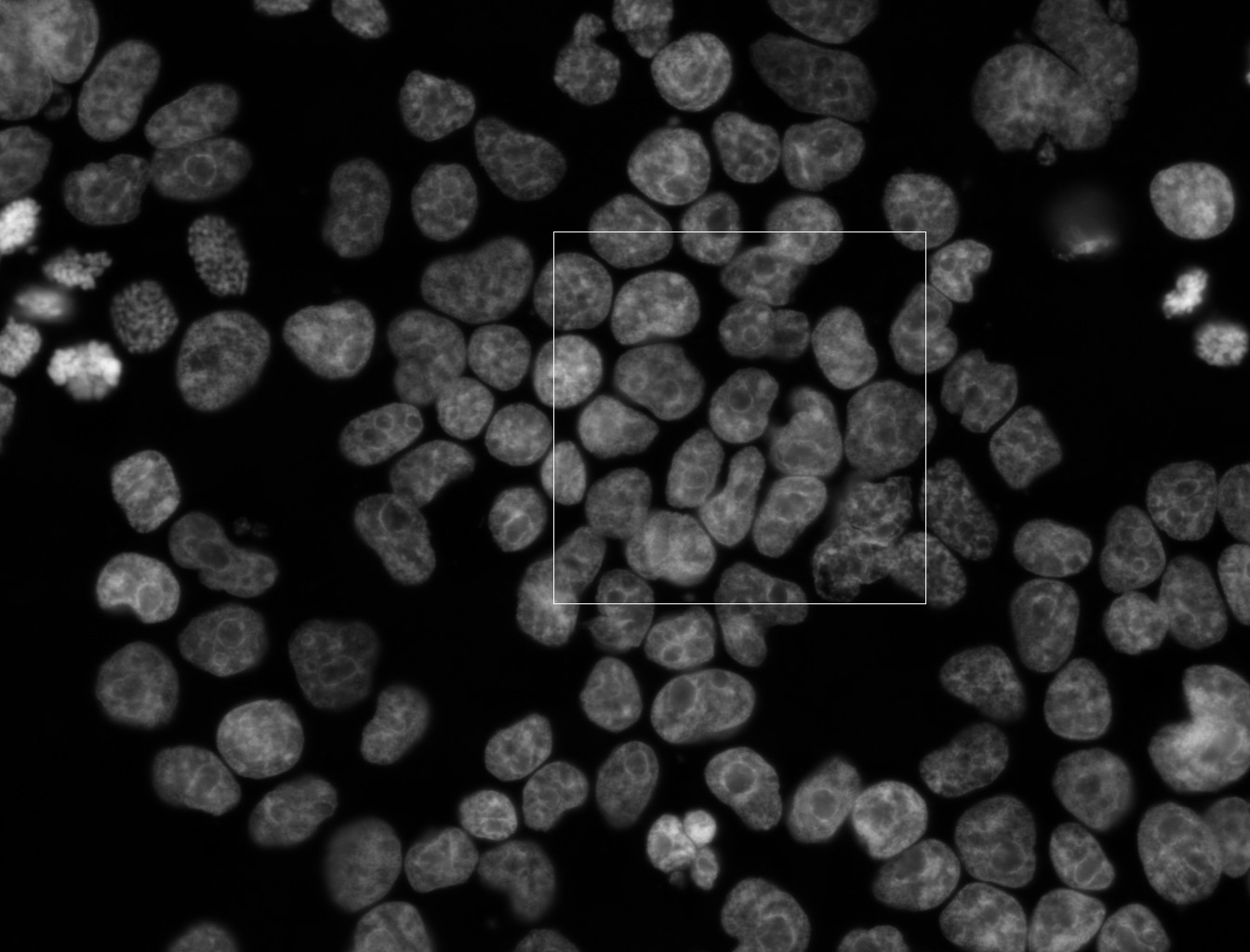

Supplement: Supplementary file 3 — Source data Fig. 1 [file 44318_2025_637_MOESM3_ESM.zip › Figure 1/1A/uninfected.tif]

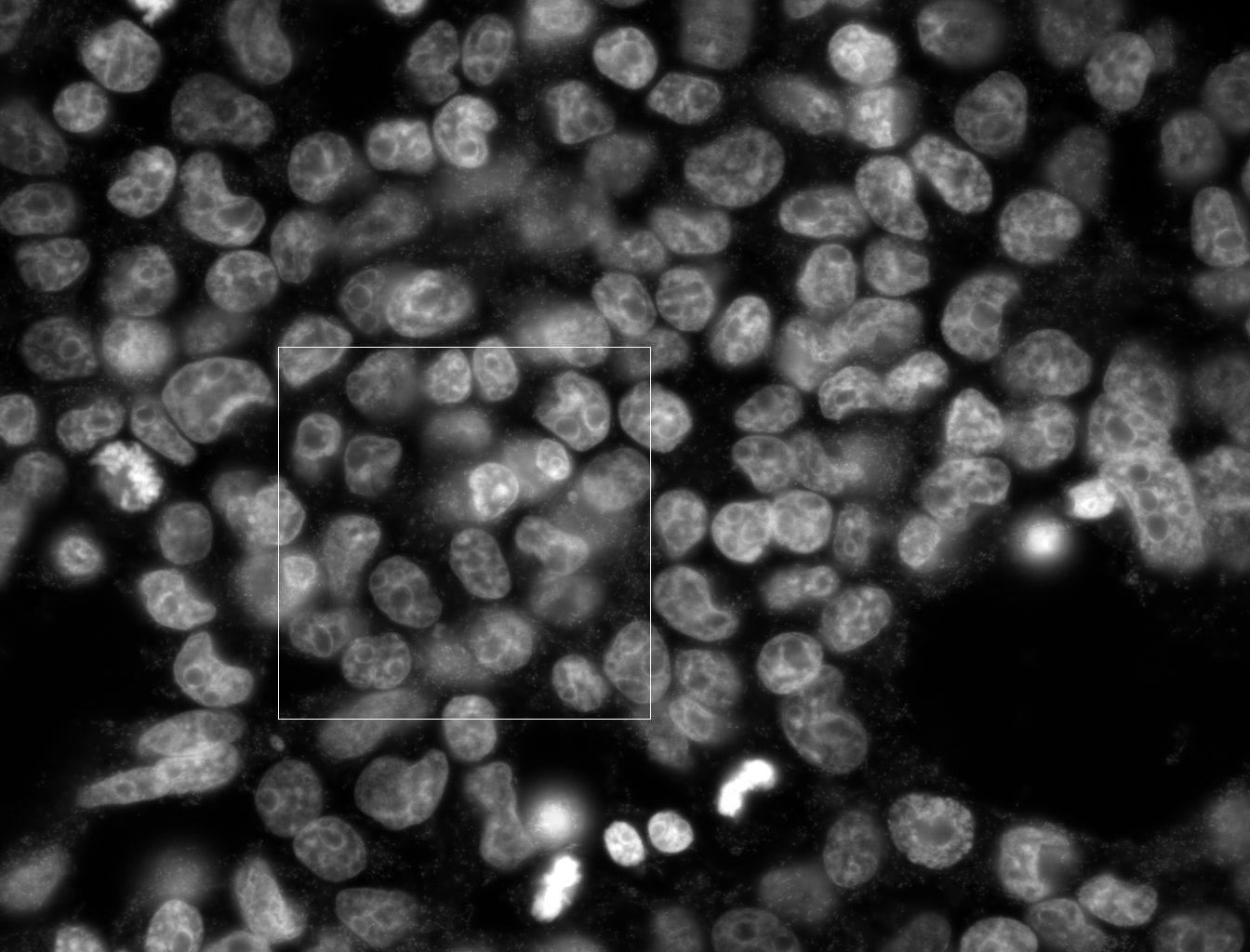

Supplement: Supplementary file 3 — Source data Fig. 1 [file 44318_2025_637_MOESM3_ESM.zip › Figure 1/1C/Listeria WT.tif]

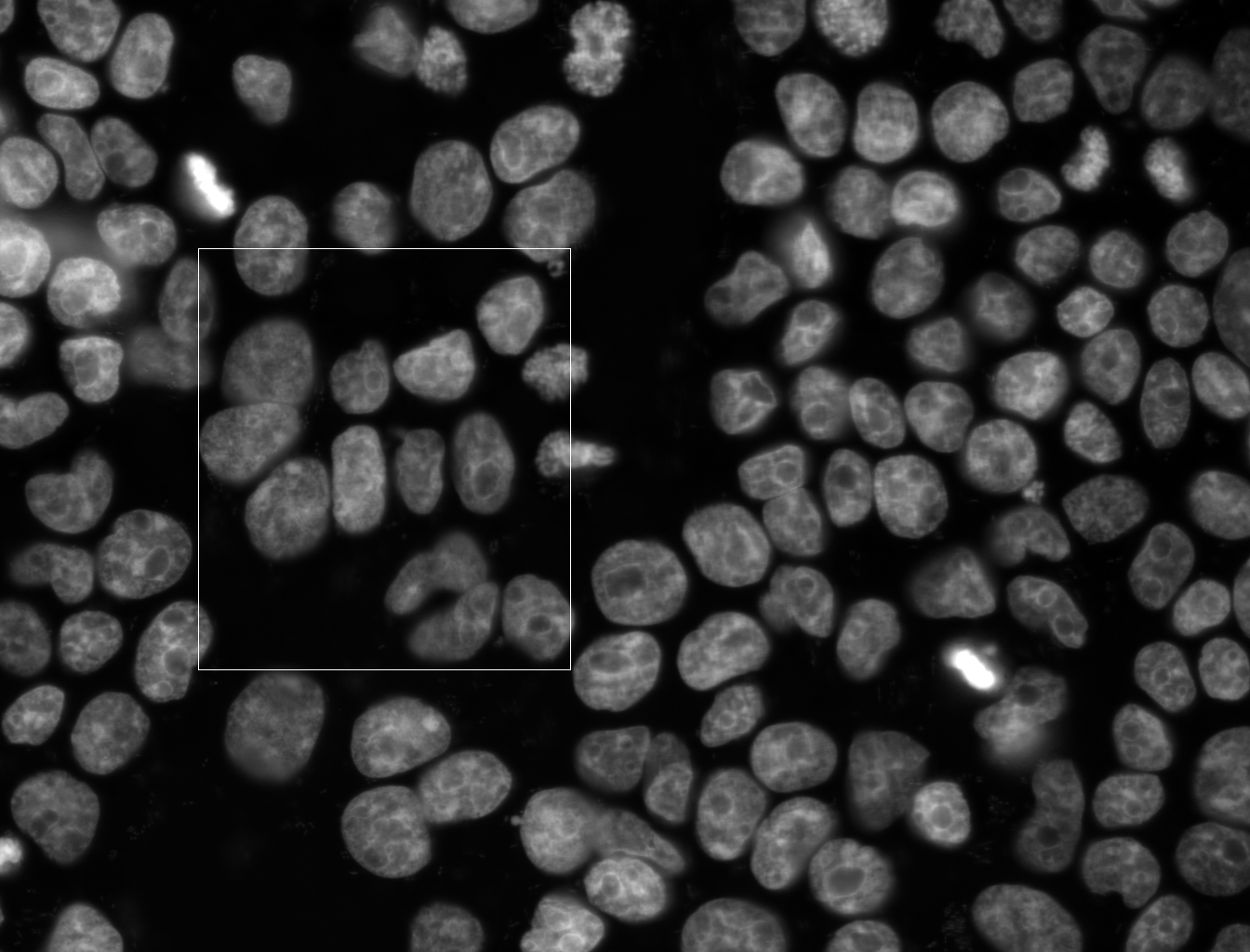

Supplement: Supplementary file 3 — Source data Fig. 1 [file 44318_2025_637_MOESM3_ESM.zip › Figure 1/1C/Listeria hly.tif]

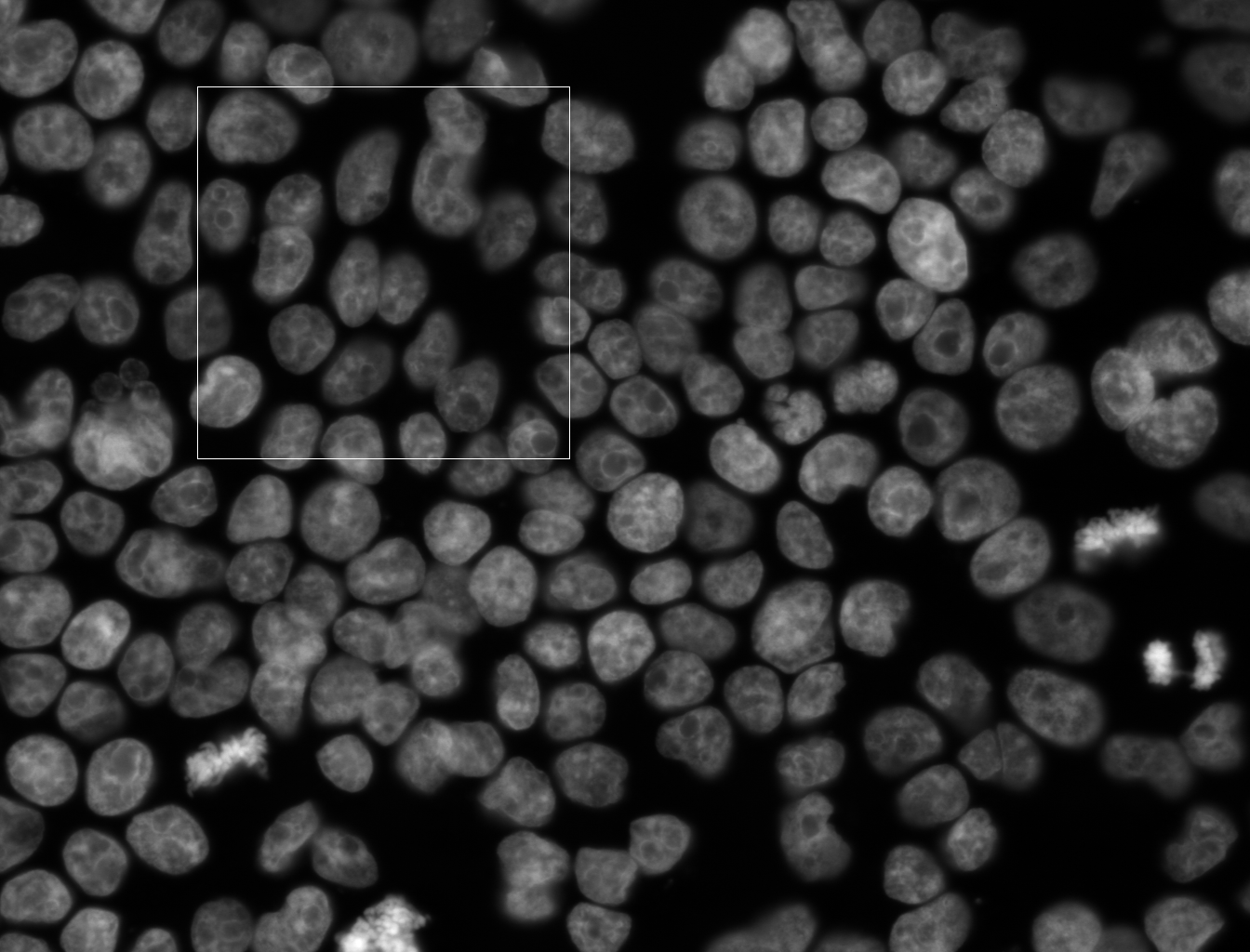

Supplement: Supplementary file 3 — Source data Fig. 1 [file 44318_2025_637_MOESM3_ESM.zip › Figure 1/1C/uninfected.tif]

**Figure 2H**

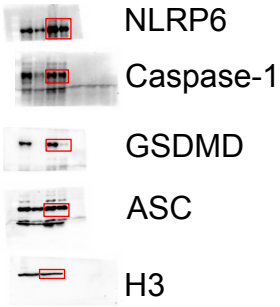

Supplement: Supplementary file 4 — Source data Fig. 2 [file 44318_2025_637_MOESM4_ESM.zip › Figure 2/2H/2H.pdf]

**Figure 2C**

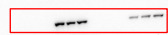

NLRP6

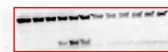

GSDMD

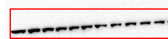

Vinculin

Supplement: Supplementary file 4 — Source data Fig. 2 [file 44318_2025_637_MOESM4_ESM.zip › Figure 2/2C/2C.pdf]

**Figure 2F**

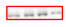

NLRP6

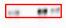

Caspase-1

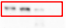

Caspase-4

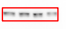

GSDMD

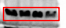

ASC

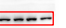

Vinculin

Supplement: Supplementary file 4 — Source data Fig. 2 [file 44318_2025_637_MOESM4_ESM.zip › Figure 2/2F/2F.pdf]

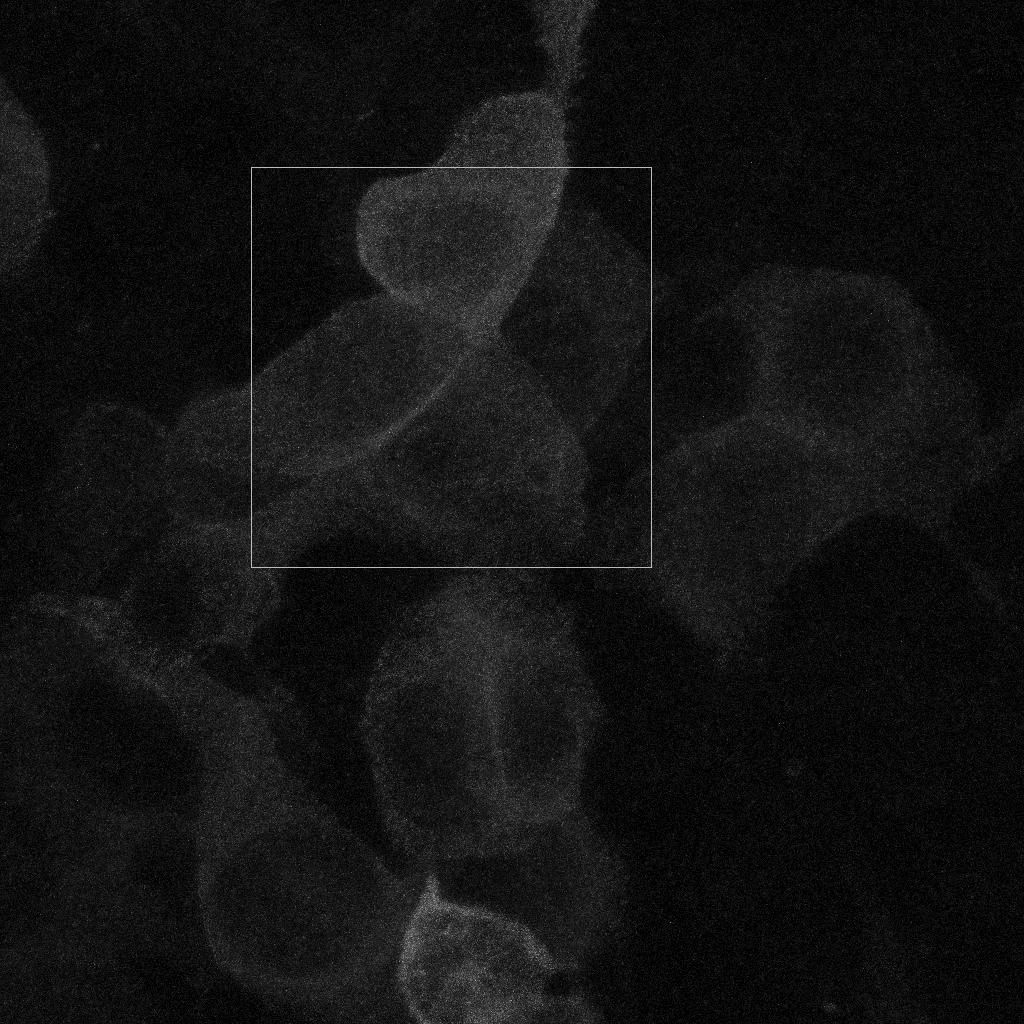

Supplement: Supplementary file 6 — Source data Fig. 4 [file 44318_2025_637_MOESM6_ESM.zip › Figure 4/4B/maxprojection BODIPY-LTA NLRP6.tif]

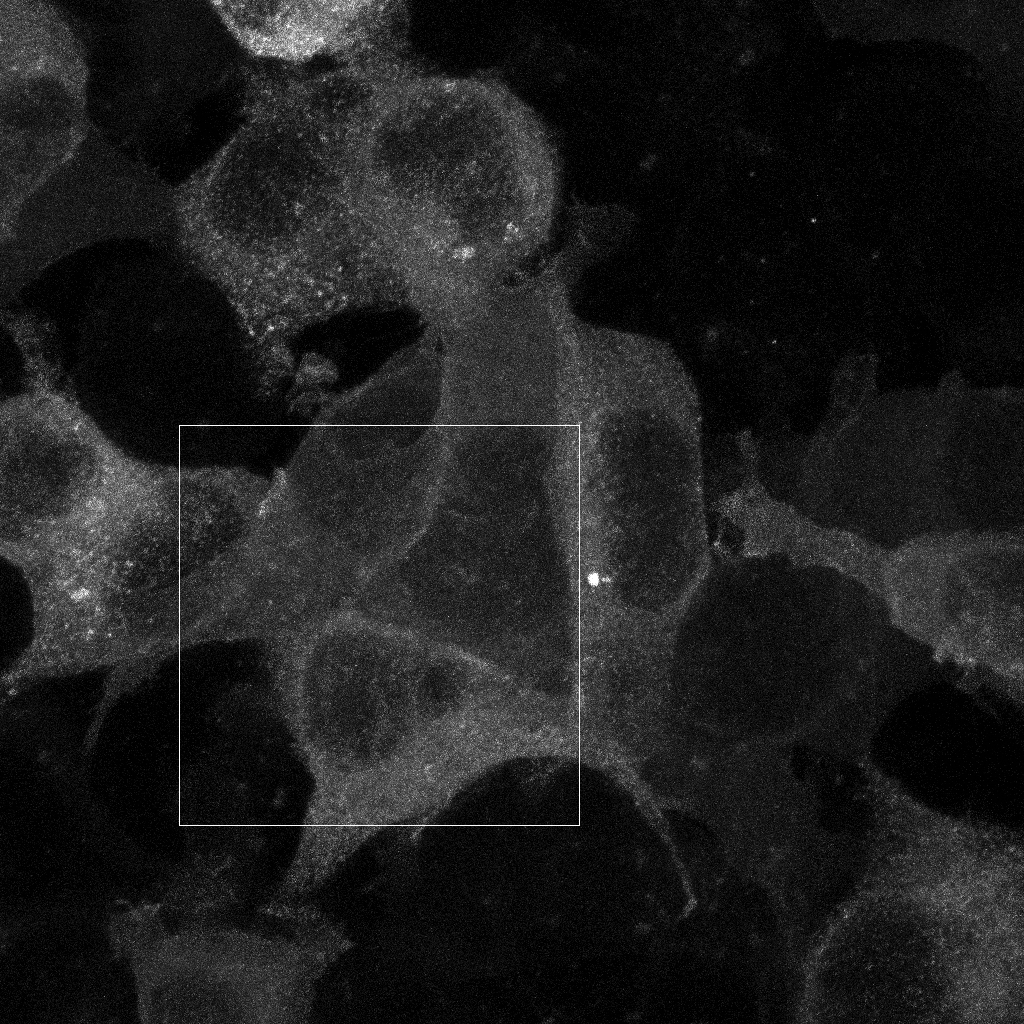

Supplement: Supplementary file 6 — Source data Fig. 4 [file 44318_2025_637_MOESM6_ESM.zip › Figure 4/4B/maxprojection fluorescein NLRP6-mCherry.tif]

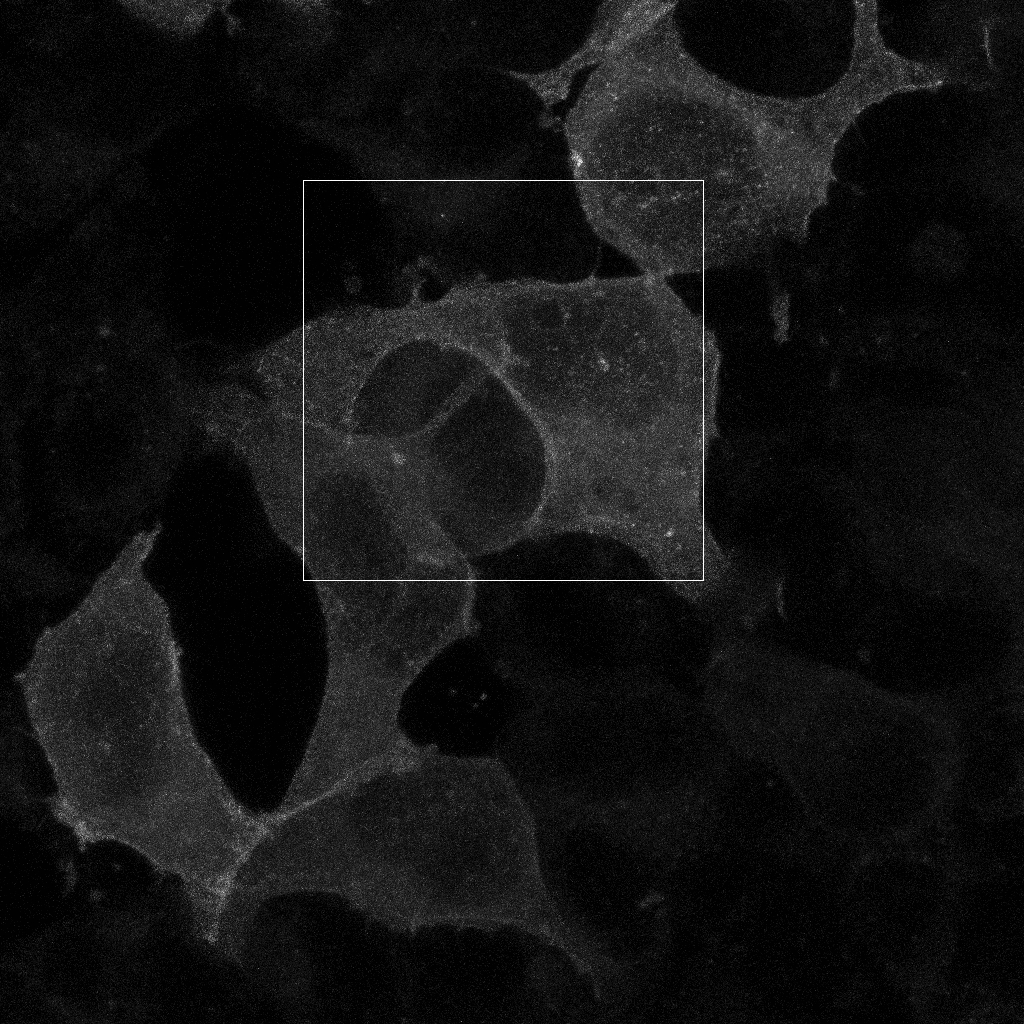

Supplement: Supplementary file 6 — Source data Fig. 4 [file 44318_2025_637_MOESM6_ESM.zip › Figure 4/4B/maxprojection poly(IC)-fluorescein NLRP6-mCherry.tif]

Figure 5B

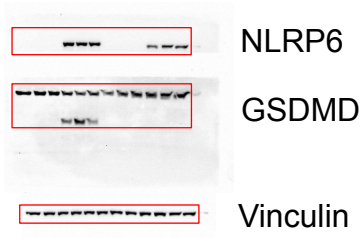

Supplement: Supplementary file 7 — Source data Fig. 5 [file 44318_2025_637_MOESM7_ESM.zip › Figure 5/5B/5B.pdf]

Figure 6B

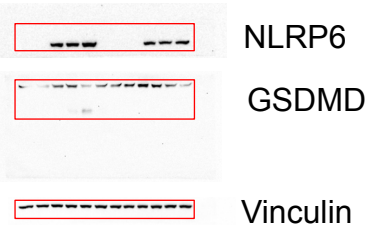

Supplement: Supplementary file 8 — Source data Fig. 6 [file 44318_2025_637_MOESM8_ESM.zip › Figure 6/6B/6B.pdf]
