# Supplementary material for: The NLRP6 inflammasome is activated by sterile or pathogen-induced endolysosomal damage
Source: EMBO J. 2025 Nov 20;45(1):30–63. doi: 10.1038/s44318-025-00637-4 (PMC12759077; doi:10.1038/s44318-025-00637-4)
Supplement: Supplementary file 9 — Expanded View Figures [file 44318_2025_637_MOESM9_ESM.pdf]

Expanded View Figures

Figure EV1. NLRP6 detects the entry of *L. monocytogenes* into the host cell cytosol.

(**A**) Flow cytometry gating strategy used to quantify ASC speck formation. (**B**) ASC speck formation quantified by flow cytometry and corresponding NLRP6 expression of GFP-ASC^{tg} HEK293T cells transfected with hNLRP6 DNA at indicated concentrations for 24 h. (**C**) ASC speck formation in hNLRP6-expressing GFP-ASC^{tg} HEK293T cells infected with *L. monocytogenes* strain EGD or 10403S, *S. aureus* or *S.* Typhimurium for 6 h. (**D**) ASC speck formation and corresponding NLRP6 expression of GFP-ASC^{tg} HEK293T cells transfected with mNLRP6 DNA at indicated concentrations for 24 h (**E**). Representative images of actin positive, intracellular *Listeria* after 1 h of infection in HEK293T cells left untreated or treated with bafilomycin A1 (bafA) for 2 h and infected with WT or Δ*hly L. monocytogenes* EGD for 6 h. *Listeria* were stained by immunofluorescence and Actin was stained with CellMask Actin Stain. Actin colocalization is indicated by arrowheads (**F**). Propidium iodide (PI) uptake of HEK293T cells after treatment with purified Listeriolysin O at indicated concentrations over time (**G**). Percentage of cells with permeabilized membranes among cells containing an ASC speck, hNLRP6-expressing GFP-ASC^{tg} HEK293T cells infected with *L. monocytogenes* EGD for 4, 6 or 8 h as measured by flow cytometry LIVE/DEAD staining. Graphs show means ± SD from at three pooled independent experiments, except panel F, which is representative of two independent experiments. Images are representative of three independent experiments, immunoblots are representative of two independent experiments. Scale bar represents 1 µm. Statistics used: one-way ANOVA with Dunnett's multiple comparisons.

 © The Author(s)

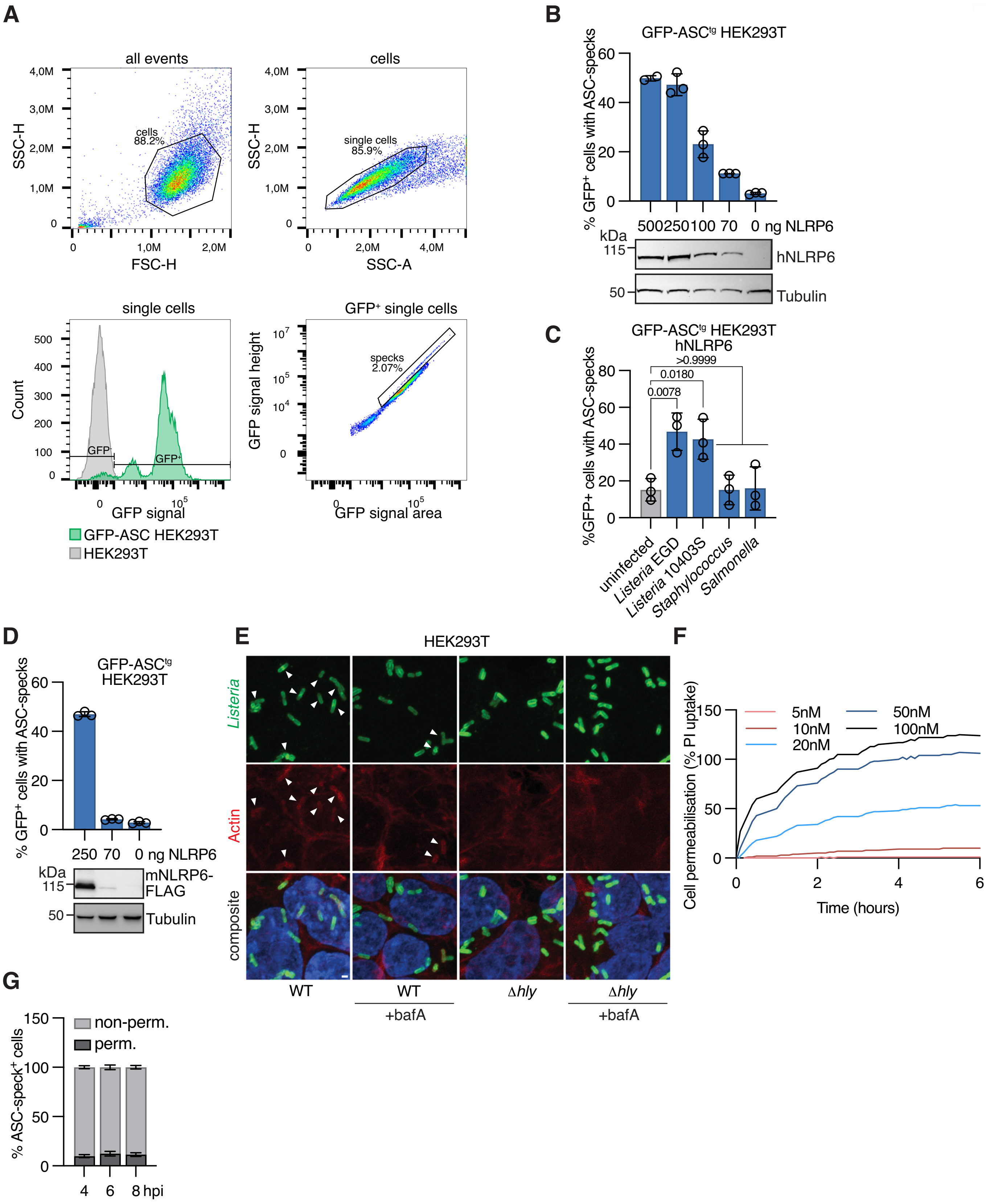
A
all events
cells 88.2%
SSC-H
FSC-H
cells
single cells 85.9%
SSC-A
single cells
Count
GFP-
GFP+
GFP signal
GFP-ASC HEK293T
HEK293T
GFP+ single cells
specks 2.07%
GFP signal height
GFP signal area
B
GFP-ASCtg HEK293T
% GFP+ cells with ASC-specks
500 250 100 70 0 ng NLRP6
kDa
hNLRP6
Tubulin
C
GFP-ASCtg HEK293T hNLRP6
>0.9999
0.0180
0.0078
%GFP+ cells with ASC-specks
uninfected
Listeria EGD
Listeria 10403S
Staphylococcus
Salmonella
D
GFP-ASCtg HEK293T
% GFP+ cells with ASC-specks
250 70 0 ng NLRP6
kDa
mNLRP6-FLAG
Tubulin
E
HEK293T
Listeria
Actin
composite
WT
WT
+bafA
Δhly
Δhly
+bafA
F
Cell permeabilisation (% PI uptake)
5nM
10nM
20nM
50nM
100nM
Time (hours)
G
non-perm.
perm.
% ASC-speck+ cells
4 6 8 hpi

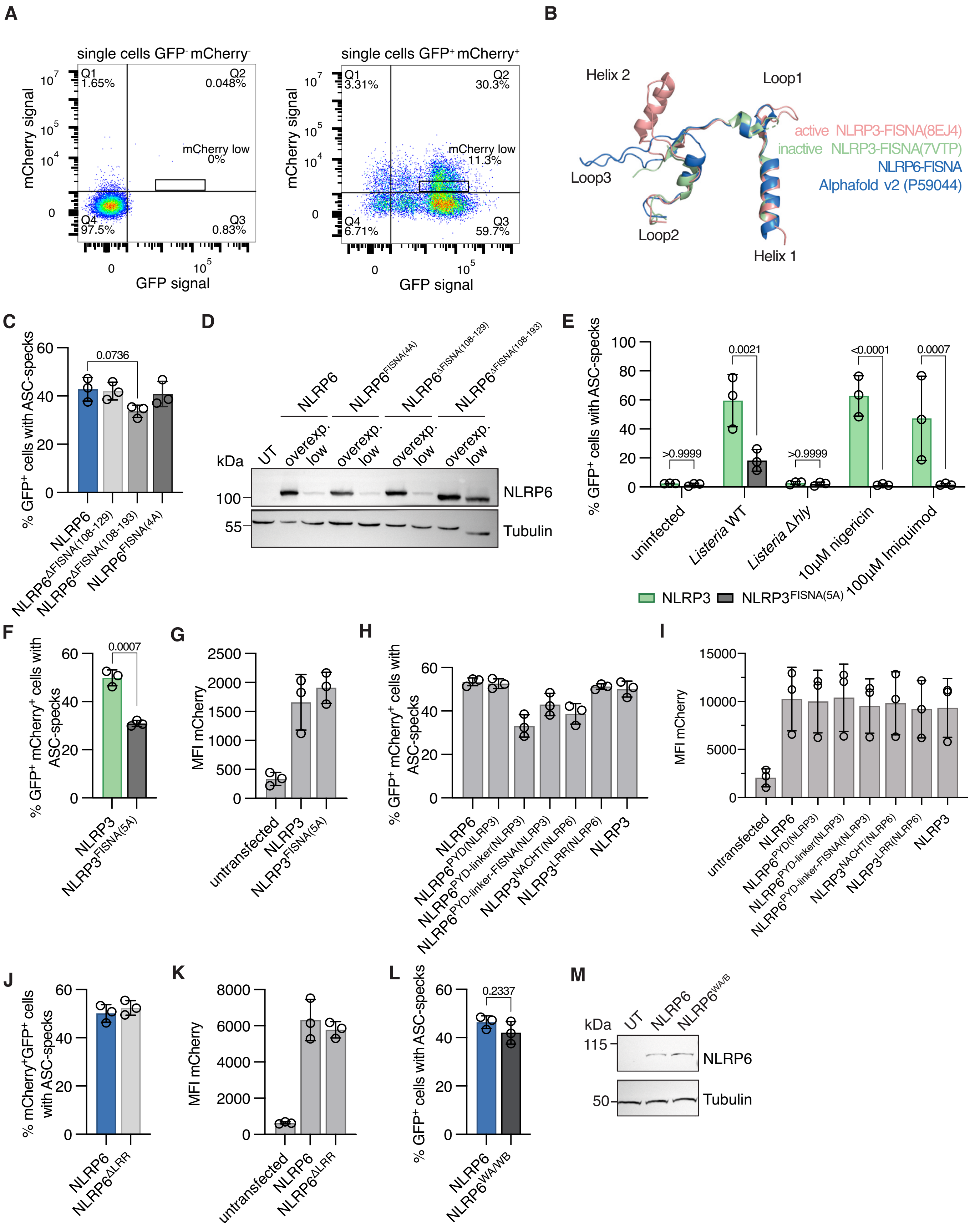
A
single cells GFP- mCherry-
single cells GFP+ mCherry+
mCherry signal
GFP signal
Q1 1.65%
Q2 0.048%
Q3 0.83%
Q4 97.5%
mCherry low 0%
Q1 3.31%
Q2 30.3%
Q3 59.7%
Q4 6.71%
mCherry low 11.3%
B
Helix 2
Loop1
Loop3
Loop2
Helix 1
active NLRP3-FISNA(8EJ4)
inactive NLRP3-FISNA(7VTP)
NLRP6-FISNA
Alphafold v2 (P59044)
C
% GFP+ cells with ASC-specks
0.0736
NLRP6
NLRP6ΔFISNA(108-129)
NLRP6ΔFISNA(108-193)
NLRP6FISNA(4A)
D
kDa
UT
overexp.
low
NLRP6
NLRP6FISNA(4A)
NLRP6ΔFISNA(108-129)
NLRP6ΔFISNA(108-193)
100
55
NLRP6
Tubulin
E
% GFP+ cells with ASC-specks
>0.9999
0.0021
>0.9999
<0.0001
0.0007
uninfected
Listeria WT
Listeria Δhly
10µM nigericin
100µM Imiquimod
NLRP3
NLRP3FISNA(5A)
F
% GFP+ mCherry+ cells with ASC-specks
0.0007
NLRP3
NLRP3FISNA(5A)
G
MFI mCherry
untransfected
NLRP3
NLRP3FISNA(5A)
H
% GFP+ mCherry+ cells with ASC-specks
NLRP6
NLRP6PYD(NLRP3)
NLRP6PYD-linker(NLRP3)
NLRP6PYD-linker-FISNA(NLRP3)
NLRP3NACHT(NLRP6)
NLRP3LRR(NLRP6)
NLRP3
I
MFI mCherry
untransfected
J
% mCherry+GFP+ cells with ASC-specks
NLRP6
NLRP6ΔLRR
K
MFI mCherry
untransfected
L
% GFP+ cells with ASC-specks
0.2337
NLRP6
NLRP6WA/WB
M
kDa
UT
NLRP6
NLRP6WA/B
115
50
NLRP6
Tubulin

Figure EV2. Molecular characterization of the NLRP6 receptor.

(**A**) Flow cytometry gating strategy used for mCherry-tagged NLRs. (**B**) Structural alignment of the FISNA domain of active and inactive NLRP3 (8EJ4 and 7VTP) with the NLRP6-FISNA predicted by Alphafold v2 (P59044). (**C**) Flow cytometry-based quantification of ASC speck formation in GFP-ASC^{tg} HEK293T cells overexpressing the indicated NLRP6 proteins at 250 ng DNA/well (**D**). Immunoblot showing expression levels of constructs depicted in C expressed at either 250 ng/well (overexp.) or 80 ng/well (low). Tubulin serves as loading control. (**E**) Flow cytometry-based quantification of ASC speck formation in GFP-ASC^{tg} HEK293T cells, expressing the indicated NLRP3-mCherry proteins, infected with WT or Δ*hly L. monocytogenes* for 6 h or treated with 10 µM nigericin or 100 µM imiquimod for 1 or 6 h, respectively (**F**). Flow cytometry-based quantification of ASC speck formation in GFP-ASC^{tg} HEK293T cells overexpressing the indicated NLRP3-mCherry proteins at 250 ng DNA/well (**G**). Mean fluorescence intensity (MFI) of NLRP3-mCherry in GFP-ASC^{tg} HEK293T cells expressing indicated NLRP3-mCherry proteins in E. For untransfected cells, this is the MFI of all single cells. For cells expressing mCherry-NLRP3 this is the MFI of cells in the mCherry low gate indicated in (**D**). (**H**) Flow cytometry-based quantification of ASC speck formation in GFP-ASC^{tg} HEK293T cells, overexpressing the indicated NLRP3-6-mCherry chimeric proteins at 250 ng DNA/well. (**I**) Mean fluorescence intensity (MFI) of NLRP3-6-mCherry chimeric proteins in GFP-ASC^{tg} HEK293T cells transfected at 80 ng DNA/well, except for chimeras NLRP3$^{NACHT(NLRP6)}$ and NLRP3$^{LRR(NLRP6)}$ which were transfected at 200 and 150 ng/well, respectively. For untransfected cells, this is the MFI of all single cells. For cells expressing mCherry-NLR chimeras this is the MFI of cells in the mCherry low gate indicated in (**D**). (**J**) Flow cytometry-based quantification of ASC speck formation in GFP-ASC^{tg} HEK293T cells, overexpressing the indicated NLRP6 proteins at 250 ng DNA/well. (**K**) Mean fluorescence intensity (MFI) of indicated NLRP6-mCherry proteins in GFP-ASC^{tg} HEK293T cells transfected at 80 ng DNA/well. For untransfected cells, this is the MFI of all single cells. For cells expressing mCherry-NLRP6 this is the MFI of cells in the mCherry low gate indicated in (**D**). (**L**) Flow cytometry-based quantification of ASC speck formation in GFP-ASC^{tg} HEK293T cells overexpressing the indicated NLRP6 proteins at 250 ng DNA/well (**M**). Immunoblot showing expression levels of indicated NLRP6 proteins in GFP-ASC^{tg} HEK293T transfected at 80 ng/well. Tubulin serves as loading control. Graphs show mean ± SD from three independent experiments. Immunoblots are representative of at least two independent experiments. Statistics used: one-way ANOVA with Dunett's multiple comparisons test (**B**), two-way ANOVA with Šídák's multiple comparisons test (**E**), unpaired *t* test (**F**, **L**).

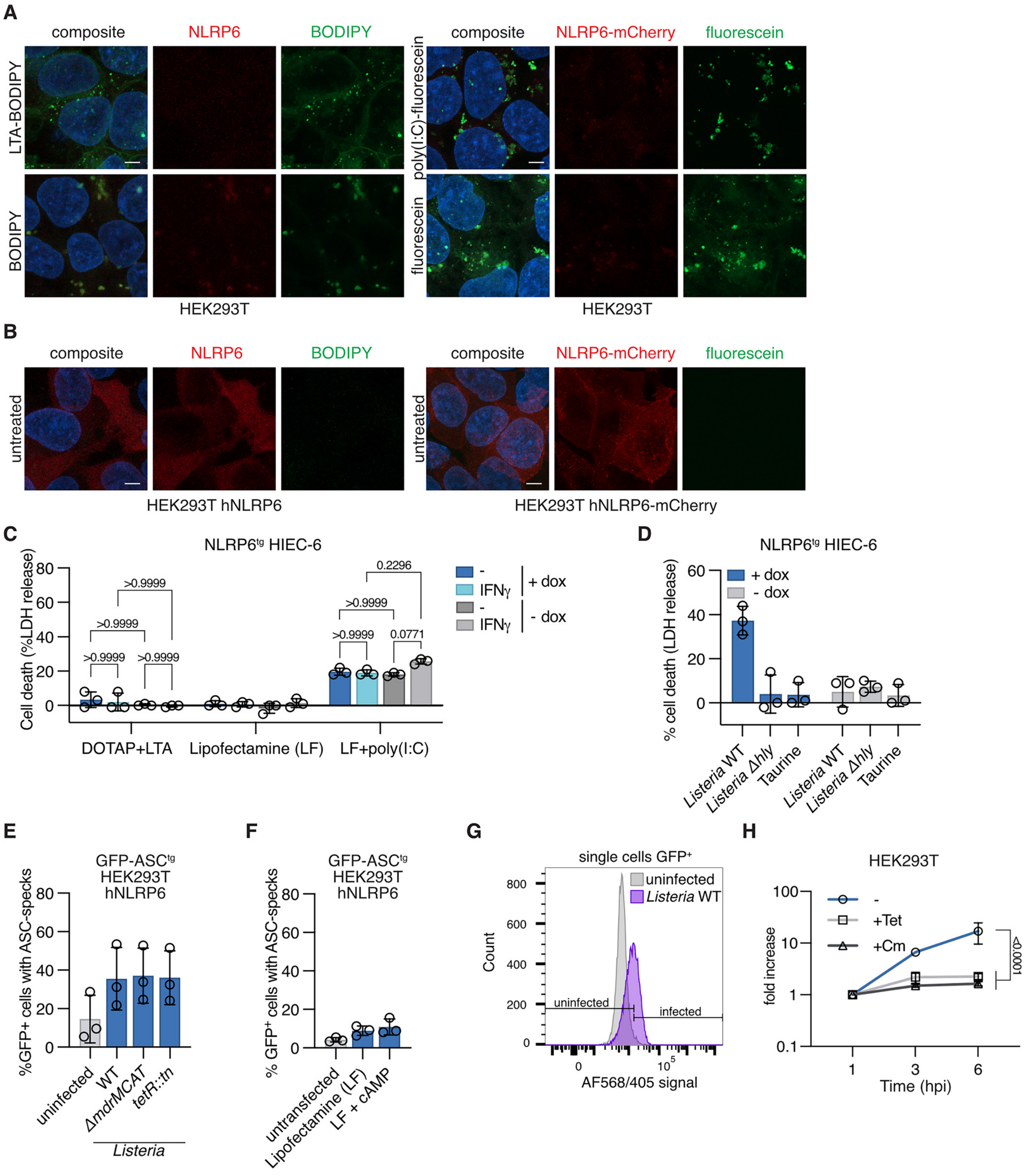
A
composite
NLRP6
BODIPY
LTA-BODIPY
BODIPY
HEK293T
composite
NLRP6-mCherry
fluorescein
poly(I:C)-fluorescein
fluorescein
HEK293T
B
composite
NLRP6
BODIPY
untreated
HEK293T hNLRP6
composite
NLRP6-mCherry
fluorescein
untreated
HEK293T hNLRP6-mCherry
C
NLRP6tg HIEC-6
Cell death (%LDH release)
DOTAP+LTA
Lipofectamine (LF)
LF+poly(I:C)
>0.9999
0.2296
0.0771
- IFNγ + dox
- IFNγ - dox
D
NLRP6tg HIEC-6
% cell death (LDH release)
+ dox
- dox
Listeria WT
Listeria Δhly
Taurine
E
GFP-ASCtg HEK293T hNLRP6
%GFP+ cells with ASC-specks
uninfected
WT
ΔmdrMCAT
tetR::tn
Listeria
F
GFP-ASCtg HEK293T hNLRP6
% GFP+ cells with ASC-specks
untransfected
Lipofectamine (LF)
LF + cAMP
G
single cells GFP+
uninfected
Listeria WT
Count
infected
AF568/405 signal
H
HEK293T
fold increase
-
+Tet
+Cm
<0.0001
Time (hpi)

 © The Author(s)

Figure EV3. NLRP6 activation does not require bacterial PAMPs, but viable cytosolic bacteria.

(**A**) Representative maximum projection confocal micrographs after transfection of HEK293T cells with LTA-BODIPY (7.5 µg/300,000), equivalent volume BODIPY only, poly(I:C)-fluorescein (250 ng/300,000 cells) or equivalent volume fluorescein only for 6 h. (**B**) Representative maximum projection confocal micrographs of hNLRP6- or hNLRP6-mCherry-expressing HEK293T cells left untreated. (**C**) LDH release from NLRP6-WT^{tg} HIEC-6 cells induced or not with 1 µg/ml doxycycline and primed or not with 10 ng/mL IFNγ overnight and transfected with Lipofectamine (LF) only or transfected with LTA or poly(I:C) for 8 h. (**D**) LDH release from NLRP6-WT^{tg} HIEC-6 cells induced or not with 1 µg/ml doxycycline overnight and infected with WT or Δ*hly L. monocytogenes* EGD for 8 h or treated with 70 mM Taurine. (**E**) Flow cytometry-based quantification of ASC speck formation in hNLRP6-expressing GFP-ASC^{tg} HEK293T cells infected with the indicated strains of *L. monocytogenes* 10403S for 8 h. (**F**) ASC speck formation in hNLRP6-expressing GFP-ASC^{tg} HEK293T cells mock treated or transfected with c-di-AMP for 24 h. (**G**) Flow cytometry gating strategy for antibody-stained *Listeria*-infected cells. (**H**) Replication of WT *L. monocytogenes* EGD over time in HEK293T cells in the presence of tetracycline (Tet) or chloramphenicol (Cm) added after 45 min of infection. Graphs show means ± SD from three independent experiments. Graphs (**C**, **D**) show mean ± SD from three pooled experiments with three technical replicates each. Each data point represents the mean of one experiment. Images are representative of three independent experiments. Scale bars represent 5 µm. Statistics used: two-way ANOVA with Šídák's multiple comparisons test.

© The Author(s)

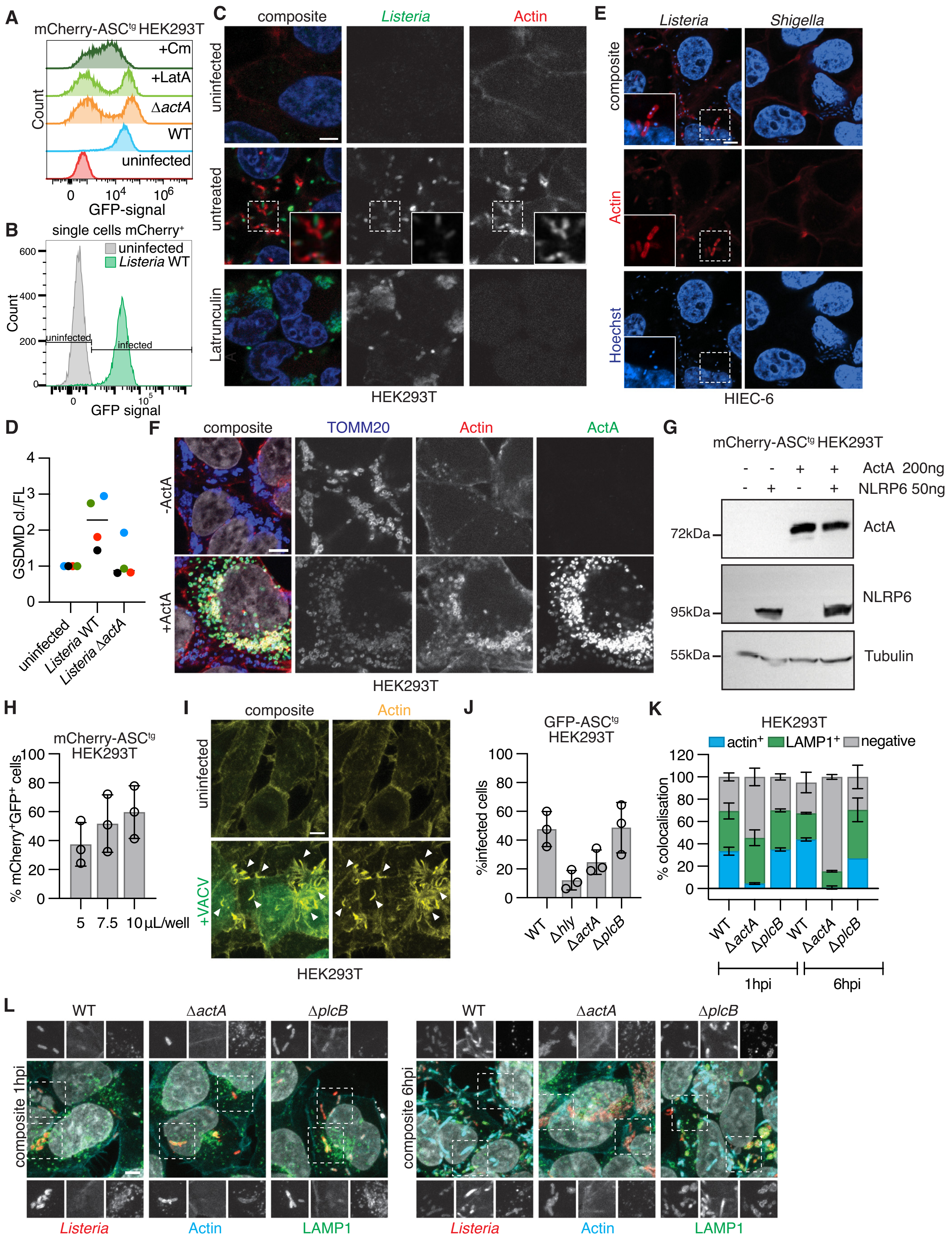
A
mCherry-ASCtg HEK293T
+Cm
+LatA
ΔactA
WT
uninfected
Count
0
GFP-signal
B
single cells mCherry+
uninfected
Listeria WT
Count
600
400
200
0
uninfected
infected
GFP signal
C
composite
Listeria
Actin
uninfected
untreated
Latrunculin
HEK293T
E
Listeria
Shigella
composite
Actin
Hoechst
HIEC-6
D
GSDMD cl./FL
4
3
2
1
0
uninfected
Listeria WT
Listeria ΔactA
F
composite
TOMM20
Actin
ActA
-ActA
+ActA
HEK293T
G
mCherry-ASCtg HEK293T
ActA 200ng
NLRP6 50ng
72kDa
95kDa
55kDa
ActA
NLRP6
Tubulin
H
mCherry-ASCtg HEK293T
% mCherry+GFP+ cells
100
80
60
40
20
0
5
7.5
10µL/well
I
composite
Actin
uninfected
+VACV
HEK293T
J
GFP-ASCtg HEK293T
%infected cells
100
80
60
40
20
0
WT
Δhly
ΔactA
ΔplcB
K
HEK293T
actin+
LAMP1+
negative
% colocalisation
120
100
80
60
40
20
0
WT
ΔactA
ΔplcB
WT
ΔactA
ΔplcB
1hpi
6hpi
L
WT
ΔactA
ΔplcB
WT
ΔactA
ΔplcB
composite 1hpi
composite 6hpi
Listeria
Actin
LAMP1
Listeria
Actin
LAMP1

 © The Author(s)

Figure EV4. NLRP6 recognizes bacterial cell-to-cell spread.

(**A**) Distribution of GFP signal as analyzed by FACS in hNLRP6-expressing mCherry-ASC^{tg} HEK293T cells, infected with GFP-expressing WT or Δ*actA L. monocytogenes* EGD for 6 h, or infected with GFP-expressing WT *L. monocytogenes* EGD and treated with Latrunculin A (LatA) or chloramphenicol (Cm) after 45 min of infection. (**B**) Flow cytometry gating strategy for GFP-tagged *Listeria*-infected cells. (**C**) Maximum projection confocal micrographs of actin tail formation by GFP-expressing WT *L. monocytogenes* EGD, treated with Latrunculin A (LatA) and imaged at 6hpi. Actin was stained with CellMask Actin Stain and regions of insets are marked by dashed boxes. (**D**) Quantification of GSDMD cleavage from western blots of NLRP6-WT^{tg} HIEC-6 induced with 1 µg/mL doxycycline overnight and then infected, or not, with *Listeria* for 8 h. Each color represents one biological replicate. Line represents the median. (**E**) Maximum projection micrographs of actin tail formation by *L. monocytogenes* EGD or *S. flexneri* expressing AfaI after 6 h of infection in HIEC-6, stained for Actin with CellMask Actin Stain. Arrowheads indicate actin-tails. (**F**) Maximum projection confocal micrograph of HEK293T cells exogenously expressing ActA or not, stained for ActA and TOMM20 by immunofluorescence and Actin was stained with CellMask Actin Stain. (**G**) Immunoblot for expression of hNLRP6 and ActA in mCherry-ASC^{tg} HEK293T cells expressing hNLRP6 or hNLRP6 and ActA. (**H**) Percentage of vaccinia-infected (GFP-positive) hNLRP6-expressing mCherry-ASC^{tg} HEK293T cells, infected with indicated volumes of vaccinia virus at 4.7e10 pfu/mL for 8 h. (**I**) Actin tails formed in vaccinia virus infected (GFP-positive) HEK293T cells. Actin was stained with CellMask Actin Stain and actin tails indicated by arrowheads. (**J**) Percentage of infected cells in hNLRP6-expressing mCherry-ASC^{tg} HEK293T cells, infected with WT, Δ*hly*, Δ*actA* and Δ*plcB L. monocytogenes* 10403S for 6 h. Intracellular *Listeria* were stained by immunofluorescence and analyzed by flow cytometry. (**K**, **L**) Quantification and representative maximum projection confocal micrographs of intracellular *Listeria* colocalizing with Actin or the lysosomal marker LAMP1 in HEK293T cells infected with WT, Δ*act* and Δ*plcB L. monocytogenes* 10403S after 1 and 6hpi. Inserts are marked by dotted squares. *Listeria* and LAMP1 were stained by immunofluorescence and for Actin CellMask Actin Stain was used. (**H**, **J**) Show mean ± SD from three independent experiments. Graph (**K**) shows means ± SD from two independent experiments. Images and blots are representative of at least two independent experiments. Scale bars represent 5 µm.

© The Author(s)

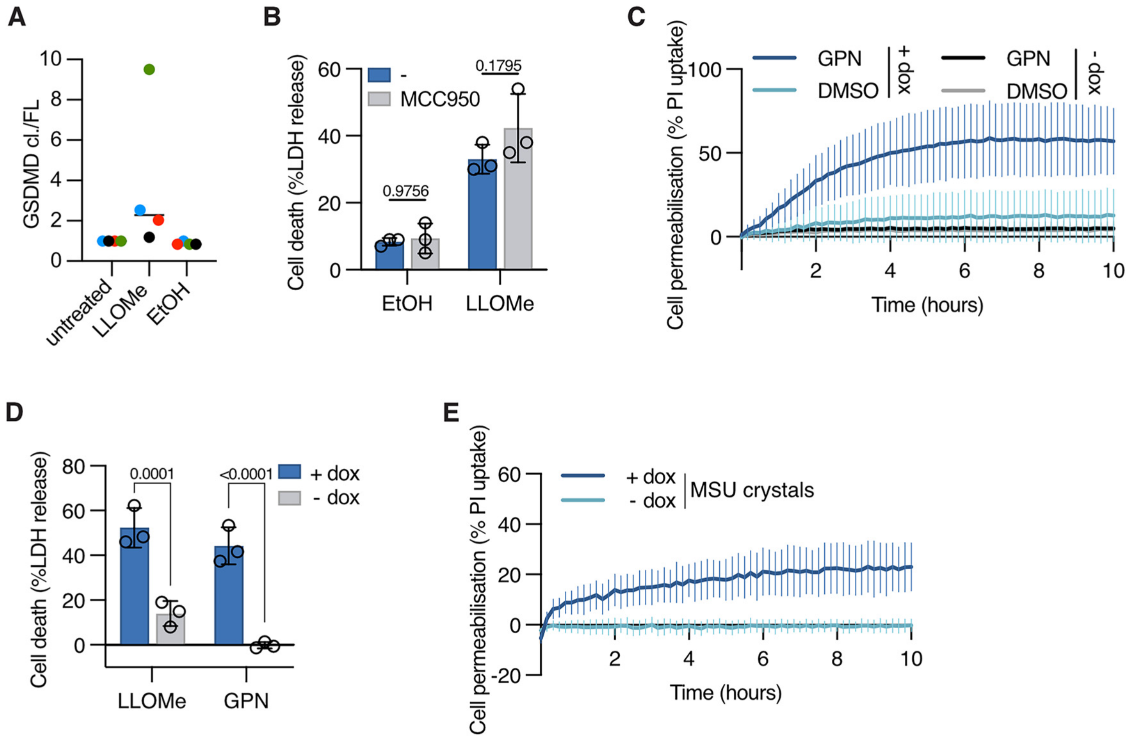

Figure EV5. Sterile endolysosomal damage is sufficient to activate NLRP6 in human intestinal epithelial cells.

(**A**) Quantification of GSDMD cleavage from Western blots of NLRP6-WT[tg] HIEC-6 induced 1 µg/mL doxycycline overnight and then treated with LLOMe or EtOH for 4 h. Each color represents one biological replicate. Line represents the median. (**B**) LDH release from NLRP6-WT[tg] HIEC-6 cells induced or not with 1 µg/ml doxycycline overnight, subsequently treated or not with 10 µM MCC950 30 min before and during treatment with LLOMe for 4 h. (**C**) Time course of propidium iodide (PI) uptake of NLRP6-WT[tg] HIEC-6 cells induced or not with 1 µg/ml doxycycline overnight and treated with 200 µM GPN or equivalent volume of DMSO (**D**). Cell death measured by LDH release from NLRP6[tg] HIEC-6 cells induced or not with 1 µg/mL doxycycline overnight and treated with 0.5 mM LLOMe or 200 µM GPN for 8 h. (**E**) Propidium iodide (PI) uptake of NLRP6-WT[tg] HIEC-6 cells induced or not with 1 µg/ml doxycycline overnight and treated with 100 µM MSU crystals for 10 h. Graphs show the mean ± SD of three independent experiments with three technical replicates each, respectively (**B–E**). Each data point shows the mean of one experiment. Statistics used in graphs (**B**, **D**): two-way ANOVA with Šídák's multiple comparisons.

 © The Author(s)
